# Clinical Evaluation of a Non-inflatable Visual Laryngeal Mask Airway: A Prospective Service Assessment in Elective and Difficult Airway Management

**DOI:** 10.7759/cureus.108699

**Published:** 2026-05-12

**Authors:** Sabrina Migliorelli, Giuseppe Pascarella, Alessandro Strumia, Francesca Claps, Felice E Agrò

**Affiliations:** 1 Anesthesia and Intensive Care, Fondazione Policlinico Universitario Campus Bio-Medico, Rome, ITA

**Keywords:** airway management, difficult airway, non-inflatable cuff, supraglottic device, visual lma

## Abstract

Background: The non-inflatable visual laryngeal mask airway (V-LMA, Tuoren Medical) integrates a high-definition optical channel with an anatomically pre-shaped, non-inflatable cuff, enabling real-time visualization and potentially reducing airway trauma. This prospective service assessment aimed to provide preliminary clinical data on the performance of the V-LMA in elective anesthesia and in predicted difficult airway management.

Methods: Thirty-eight adult patients were included, of whom eight had predicted difficult airways based on an El-Ganzouri Risk Index score >4. Primary outcomes included time to ventilation, quality of visualization, and seal performance. Secondary outcomes included gastric insufflation, need for repositioning, mucosal trauma, and postoperative symptoms.

Results: Successful device placement was achieved in all patients. The mean time to effective ventilation was 28 ± 4 seconds. Glottic visualization was consistently excellent in all cases. No gastric insufflation, postoperative mucosal injury, or patient-reported discomfort was observed. Device performance remained reliable even in patients with predicted difficult airways.

Conclusion: The V-LMA enables rapid, atraumatic, and visually guided airway management, demonstrating excellent performance even in challenging airway conditions. It may serve as a useful first-line supraglottic airway device (SAD) when maintenance of oxygenation is the primary priority.

## Introduction

Supraglottic airway devices (SADs) play a central role in contemporary airway management, as they allow clinicians to establish ventilation quickly and with minimal invasiveness [1]. Traditional laryngeal mask airways (LMAs) typically rely on inflatable cuffs, a design that may increase mucosal pressure, cause nerve compression, and potentially limit safety and patient comfort [2]. Moreover, these devices do not allow visual confirmation of correct positioning, which may reduce confidence in placement, particularly in difficult airway scenarios [3-10].

The non-inflatable visual LMA (V-LMA, Tuoren Medical) was developed to address several of these limitations and incorporates a number of technical innovations [11]. The device features an anatomically pre-shaped, non-inflatable cuff designed to conform to the airway while reducing the risk of mucosal compression. An integrated high-definition visual channel enables real-time visualization during insertion and positioning, allowing immediate confirmation of correct placement. The addition of an esophageal drainage lumen enhances airway protection, while the multi-lumen configuration allows for potential guided intubation through the device when required. The system also includes a displacement alarm designed to detect loss of seal or device movement after placement.

According to the manufacturer’s technical documentation [11], these features collectively support real-time visual monitoring and atraumatic placement, making the device suitable for a range of clinical settings, including elective anesthesia, emergency airway management, and predicted difficult airways.

This prospective clinical experience evaluates the performance of this device in routine and predicted difficult airway management.

The primary aim of this prospective observational service evaluation was to assess the clinical performance of the V-LMA in terms of time to effective ventilation, quality of visualization, and airway seal adequacy. Secondary aims included assessment of gastric insufflation, need for repositioning maneuvers, mucosal trauma, and postoperative airway symptoms.

## Materials and methods

Technical report

**Figure 1:**
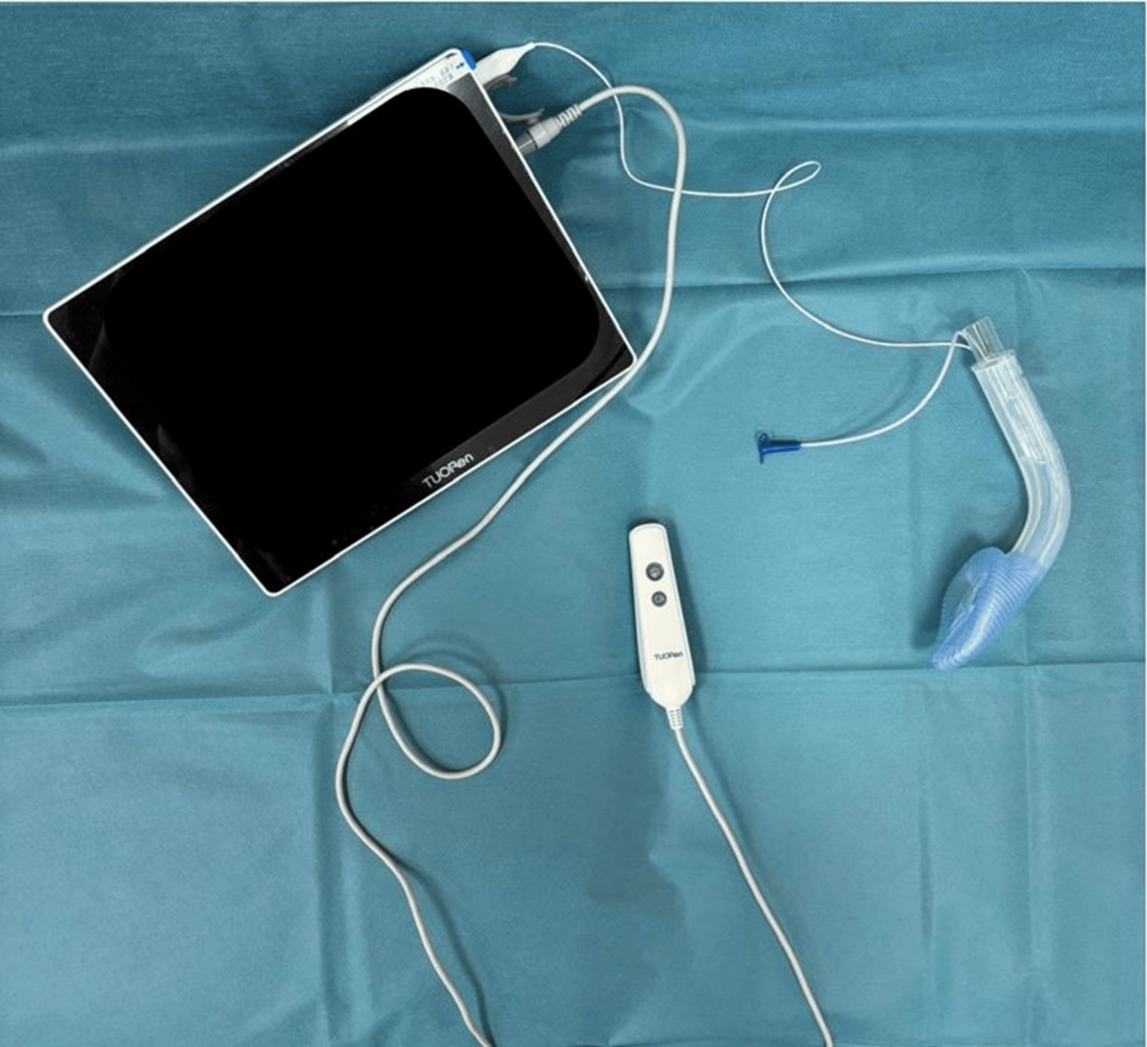
The non-inflatable visual laryngeal mask airway (Tuoren Medical) Integrated high-definition camera, multi-lumen structure, and non-inflatable anatomically curved cuff. The real-time visual feed displayed on the monitor allows direct confirmation of glottic exposure and correct positioning.

The V-LMA consists of a preformed non-inflatable cuff, a ventilation lumen, a high-definition optical channel located at the distal tip adjacent to the ventilation outlet, and an esophageal drainage lumen. The device is constructed from medical-grade thermoplastic materials designed to provide flexibility while maintaining structural integrity. The distal optical module incorporates a high-definition camera with an integrated light source, enabling continuous real-time visualization during insertion without generating clinically significant heat. According to the manufacturer’s technical documentation [11], these features may improve placement accuracy while eliminating risks associated with variable cuff pressure (Table 1, Figure 1).

**Table 1:** Key features and clinical benefits of the visual laryngeal mask airway (V-LMA)

Device feature	Clinical benefit
Non-inflatable cuff	Lower mucosal pressure, reduced trauma
High-definition optical channel	Real-time visualization
Esophageal drainage lumen	Reduced gastric insufflation
Multi-lumen design	Potential for guided intubation
Depth markings	Facilitates correct placement
Displacement alarm	Alerts to seal loss

The device is commercially available and complies with applicable medical device regulations according to the manufacturer's specifications.

Detailed engineering specifications, including exact curvature angle, internal lumen diameters, and minimum inter-incisor opening requirements, are defined by the manufacturer and may vary according to device size.

Study design

A prospective service evaluation was conducted at the Fondazione Policlinico Universitario Campus Bio-Medico of Rome. Institutional Review Board (IRB) approval was not required under institutional policies for quality-improvement initiatives conducted within standard clinical practice. The requirement for informed consent was waived in accordance with institutional policies for service evaluation studies. All patient data were anonymized.

Thirty adult patients without predicted airway difficulty and eight patients with an El-Ganzouri Risk Index (EGRI) score >4 were included. Patients with severe airway obstruction, markedly increased airway resistance, or refusal to participate were excluded.

Patients were consecutively enrolled during routine clinical practice over the study period without randomization.

No formal sample size calculation was performed, as the study was designed as an exploratory service evaluation intended to generate preliminary clinical data. The sample size was considered pragmatic and consistent with early clinical evaluations of novel airway devices.

Procedure

General anesthesia was induced with intravenous propofol (2 mg/kg), rocuronium (0.6-1.2 mg/kg), and fentanyl (1-3 mcg/kg). Standard intraoperative monitoring included electrocardiography, non-invasive blood pressure measurement, pulse oximetry, and capnography. Patients were positioned in the sniffing position prior to device insertion. Anesthesia was maintained with sevoflurane in an oxygen-air mixture according to standard clinical practice. Sevoflurane concentration was adjusted using Bispectral Index (BIS) monitoring to maintain adequate anesthetic depth. The posterior surface of the V-LMA was lubricated with a water-soluble gel before insertion. Device insertion was performed after confirmation of adequate neuromuscular blockade using neuromuscular monitoring (train-of-four ratio 0/4). The V-LMA was inserted following the natural oropharyngolaryngeal curvature until optimal positioning was achieved. Device size was selected according to manufacturer recommendations based on patient body weight and airway characteristics. Correct placement was confirmed by direct visualization through the integrated optical channel and by the presence of a stable end-tidal CO₂ (EtCO₂) waveform. Ventilation was performed using positive-pressure ventilation according to standard anesthetic practice. Successful positioning was defined as effective positive-pressure ventilation, a stable EtCO₂ waveform, absence of clinically significant air leak, direct visualization of the laryngeal inlet, and no requirement for repositioning maneuvers following first-attempt insertion. Airway seal adequacy was clinically assessed by the absence of audible air leak and maintenance of effective ventilation without significant loss of tidal volume.

Endpoints

The evaluation focused on the following endpoints. (1) Time to effective ventilation: Adequacy of ventilation was clinically assessed based on the presence of a stable end-tidal CO₂ waveform, visible chest expansion, maintenance of peripheral oxygen saturation, and absence of clinically significant audible air leak during positive-pressure ventilation. (2) Quality of visualization through the optical channel: Visualization quality was clinically assessed based on the ability to clearly identify the laryngeal inlet and surrounding anatomical landmarks during device insertion and positioning. (3) Adequacy of airway seal: Airway seal adequacy was clinically assessed by the ability to maintain effective positive-pressure ventilation, visible chest expansion, maintenance of peripheral oxygen saturation, absence of clinically significant audible air leak or relevant tidal volume loss, and lack of need for repositioning maneuvers. A clinically significant air leak was defined as an audible leak associated with impaired positive-pressure ventilation, inadequate chest expansion, unstable or absent end-tidal CO₂ waveform, or inability to maintain effective ventilation. (4) Presence or absence of gastric insufflation: Gastric insufflation was clinically assessed by evaluating for epigastric distension and audible gastric air insufflation during positive-pressure ventilation. (5) Postoperative airway discomfort: Postoperative airway symptoms, including sore throat, dysphagia, and dysphonia, were assessed during the immediate postoperative recovery period in the post-anesthesia care unit (PACU), following recovery from anesthesia. Assessment was performed through direct patient questioning and clinical evaluation by the anesthesia team.

All outcome measures were assessed in real time during the procedure by the anesthesiologist performing the airway management. All operators were senior anesthesiologists with comparable experience in airway management, reducing inter-operator variability.

Statistical analysis

Given the exploratory and descriptive nature of this prospective observational service evaluation, only descriptive statistical analyses were performed. Continuous variables are reported as mean ± standard deviation (SD), while categorical variables are presented as counts and percentages. No comparative or inferential statistical analyses were planned.

## Results

Successful device placement on the first insertion attempt, defined as correct positioning allowing effective ventilation according to the predefined clinical criteria, was achieved in all 38 patients included in the evaluation. No rescue airway maneuvers or alternative airway devices were required. Patient demographics are summarized in Table 2.

**Table 2:** Patients’ demographics ASA: American Society of Anesthesiologists, BMI: body mass index, EGRI: El-Ganzouri Risk Index.

Variable	Value
Number of patients	38
Age (years), mean ± SD	52 ± 11
Sex (M/F)	21/17
BMI (kg/m²), mean ± SD	27.4 ± 3.2
ASA class (I/II/III)	14/19/5
Predicted difficult airways (EGRI > 4)	8

The mean time required to establish effective ventilation was 28 ± 4 seconds (standard deviation (SD)) (Table 3). Visualization of the laryngeal inlet and surrounding anatomical landmarks through the integrated optical channel was consistently excellent, with all cases providing a full view of the aditus ad laryngem, allowing immediate confirmation of correct placement. Ventilation remained adequate throughout the procedure in all patients. No clinically significant air leaks were observed, and airway seal stability was maintained in every case. No episodes of gastric insufflation occurred, and no repositioning maneuvers were required after initial placement. No complications were observed during device removal. Specifically, no patients experienced postoperative sore throat, dysphagia, dysphonia, or evidence of mucosal trauma, as assessed in the PACU following recovery from anesthesia. No thermal discomfort or device overheating was reported. The results are summarized in Table 3.

**Table 3:** Clinical performance and safety outcomes of the device

Outcome	Results
Time to ventilation (mean ± SD)	28 ± 4 seconds
Glottic visualization (n, %)	38 (100)
Seal (n, %)	38 (100)
Gastric insufflation (n, %)	0 (0)
Postoperative symptoms (n, %)	0 (0)
Mucosal trauma (n, %)	0 (0)
Thermal discomfort or overheating (n, %)	0 (0)

Difficult airway group

Among the eight patients with predicted difficult airways, defined by an EGRI score >4, the device demonstrated consistent clinical performance. Visualization through the integrated optical channel allowed clear identification of the laryngeal inlet and surrounding anatomical landmarks in all cases, and none of the patients required rescue airway intervention. These findings support the potential utility of the V-LMA as a first-line SAD in anticipated difficult airway management.

## Discussion

This evaluation suggests that the non-inflatable V-LMA is an intuitive, reliable, and safe airway device across a broad clinical spectrum.

Previous studies have highlighted several limitations of inflatable cuff LMAs, including mucosal trauma, cuff overinflation, and malposition, which may compromise airway seal and patient safety [12,13].

In contrast, devices using non-inflatable cuffs have been reported to reduce pressure-related mucosal injury and postoperative airway discomfort and to demonstrate favorable insertion characteristics [13], supporting the design rationale of the V-LMA.

Furthermore, visual-guided airway techniques, such as videolaryngoscopy, have been shown to improve placement accuracy, increase first-pass success rates, and reduce airway-related complications, particularly in difficult airway scenarios [14].

The V-LMA extends this concept by integrating continuous visualization directly into a supraglottic airway device, thereby bridging the gap between conventional LMAs and visual-guided intubation tools. The non-inflatable cuff eliminates the need for pressure calibration and may reduce mucosal trauma, a well-documented complication of traditional LMAs. The integrated visual channel provides immediate confirmation of anatomical positioning, potentially reducing the risk of unrecognized malposition. In addition, the gastric drainage lumen enhances safety by reducing the risk of gastric insufflation. The multi-lumen configuration may also allow for guided tracheal intubation through the device under direct visual control, offering a potential advantage in the management of predicted difficult airways. However, this functionality was not specifically evaluated in the present study and requires further investigation.

The present findings should be interpreted within the context of existing SADs. Direct comparative studies with established devices are necessary to determine whether these theoretical advantages translate into clinically meaningful benefits.

Notably, the consistently high-quality visualization observed in patients with an EGRI score >4 suggests that this device may serve as a reliable first-line approach in situations where maintaining oxygenation is the primary goal. Ventilation was stabilized immediately in all cases, reflecting the device’s effectiveness even in the presence of anticipated airway difficulty.

However, this study has several limitations. The absence of a predefined sample size calculation and formal statistical power analysis limits the ability to draw definitive conclusions or detect small differences in performance. The single-center design may also limit generalizability. In addition, the absence of a comparator group precludes direct comparison with established SADs or videolaryngoscopy. As a service evaluation rather than a randomized controlled trial, causal inferences cannot be established. An additional limitation is the lack of a universally accepted definition of “effective ventilation” in airway research. In this study, effective ventilation was pragmatically defined using clinically applicable criteria, including a stable end-tidal CO₂ waveform, adequate chest expansion, and absence of significant air leak. However, alternative definitions exist in the literature. Future studies may benefit from standardized terminology frameworks such as the Universal Airway Collaboration glossary, which aims to improve consistency in airway-related definitions and outcome reporting [15]. Furthermore, no validated visualization scoring system is currently available for this device. Therefore, visualization quality was assessed descriptively in real time by the operator, based on the ability to clearly identify the laryngeal inlet and surrounding anatomical landmarks through the integrated optical channel, which may introduce subjectivity. Future studies should explore whether standardized visualization grading systems can be adapted for visual SADs. Moreover, objective ventilatory parameters such as peak airway pressure, oropharyngeal leak pressure, and tidal volume were not systematically measured, limiting quantitative assessment of seal performance and ventilation efficacy. Gastric insufflation was assessed clinically rather than using objective imaging methods such as gastric ultrasonography. Postoperative airway symptoms were also evaluated clinically and not using validated symptom scoring tools. In addition, no formal statistical reliability analysis, including inter-rater agreement or variability assessment, was performed in this exploratory study. Therefore, conclusions regarding reproducibility and reliability should be interpreted with caution.

The present study did not specifically evaluate the use of the V-LMA in morbidly obese or pregnant patients. Therefore, the safety and effectiveness of the device in these higher-risk airway populations remain uncertain and should be investigated in future dedicated studies.

Nevertheless, this study provides preliminary real-world clinical data regarding the feasibility and performance of this novel visual supraglottic airway device.

Although the present findings are encouraging, larger multicenter randomized comparative studies against established supraglottic airway devices are required to better define the clinical role, safety profile, and potential advantages of the V-LMA across different airway management scenarios.

## Conclusions

The non-inflatable V-LMA demonstrated reliable and efficient airway management, combining rapid placement with continuous visual guidance and minimal airway trauma. Across both elective procedures and predicted difficult airway situations, the device showed consistent stability and effective maintenance of ventilation and oxygenation, supporting accurate positioning and early recognition of potential issues. These features may translate into improved procedural safety and operator confidence, particularly in scenarios where airway visualization is advantageous or uninterrupted oxygen delivery is critical. Overall, the findings suggest that this device represents a promising supraglottic airway option and may be considered as a primary airway management tool in a broad range of clinical settings. Further large-scale randomized comparative studies are warranted before this device can be definitively recommended as a first-line SAD.
